# Age and Gender Differences in Emotion Recognition

**DOI:** 10.3389/fpsyg.2019.02371

**Published:** 2019-10-23

**Authors:** Laura Abbruzzese, Nadia Magnani, Ian H. Robertson, Mauro Mancuso

**Affiliations:** ^1^Tuscany Rehabilitation Clinic, Montevarchi, Italy; ^2^Adult Mental Health Service, NHS-USL Tuscany South-Est, Grosseto, Italy; ^3^Global Brain Health Institute, Trinity College Institute of Neuroscience, Trinity College Dublin, The University of Dublin, Dublin, Ireland; ^4^Physical and Rehabilitative Medicine Unit, NHS-USL Tuscany South-Est, Grosseto, Italy

**Keywords:** emotion recognition, gender differences, age differences, eye movements, cognitive functioning, satisfaction of life

## Abstract

**Background:**

Existing literature suggests that age affects recognition of affective facial expressions. Eye-tracking studies highlighted that age-related differences in recognition of emotions could be explained by different face exploration patterns due to attentional impairment. Gender also seems to play a role in recognition of emotions. Unfortunately, little is known about the differences in emotion perception abilities across lifespans for men and women, even if females show more ability from infancy.

**Objective:**

The present study aimed to examine the role of age and gender on facial emotion recognition in relation to neuropsychological functions and face exploration strategies. We also aimed to explore the associations between emotion recognition and quality of life.

**Methods:**

60 healthy people were consecutively enrolled in the study and divided into two groups: Younger Adults and Older Adults. Participants were assessed for: emotion recognition, attention abilities, frontal functioning, memory functioning and quality of life satisfaction. During the execution of the emotion recognition test using the Pictures of Facial Affects (PoFA) and a modified version of PoFA (M-PoFA), subject’s eye movements were recorded with an Eye Tracker.

**Results:**

Significant differences between younger and older adults were detected for fear recognition when adjusted for cognitive functioning and eye-gaze fixations characteristics. Adjusted means of fear recognition were significantly higher in the younger group than in the older group. With regard to gender’s effects, old females recognized identical pairs of emotions better than old males. Considering the Satisfaction Profile (SAT-P) we detected negative correlations between some dimensions (Physical functioning, Sleep/feeding/free time) and emotion recognition (i.e., sadness, and disgust).

**Conclusion:**

The current study provided novel insights into the specific mechanisms that may explain differences in emotion recognition, examining how age and gender differences can be outlined by cognitive functioning and face exploration strategies.

## Introduction

Social perception can be described as the ability to understand and react appropriately to the social signals sent out by other people vocally, facially, or through body posture (Mayer et al., 2016). The value of emotional information can exert significant effects on human performance as it drives behaviors in approaching or avoiding social interactions. As facial expressions convey a wealth of social information, their recognition interacts with various cognitive processes such as attention and memory. Specific emotional information (happy and sad faces) is associated with focused or distributed attention respectively (Srinivasan and Gupta, 2010). Global processing seems to facilitate recognition of happy faces while local processing seems to facilitate recognition of sad faces (Srinivasan and Gupta, 2011). Differences in emotion-attention interaction lead to differential effects on conscious perception of irrelevant emotional stimuli. A better identification of happy faces compared to sad faces and an inhibition of unattended sad distractors faces is detected in the condition of high processing demands placed on the perceptual system. This indicates an influence of inhibitory processes during difficult attentional tasks on perception of some distractor stimuli (Gupta and Srinivasan, 2014). Social affect cues like irrelevant distractor faces with happy or sad expression can influence cognitive control as in the case of error processing. A slowness in response immediately after an error only occurs when distractors are constituted by a happy face, suggesting the need for additional processing when positive performance feedback occurs after an error (Gupta and Deák, 2015).

Emotions interact with memory improving encoding or retrieval of emotional information (Christianson, 1992; Buchanan and Adolphs, 2003). Holistic processing plays a crucial role in face identification and in long-term memory, as it determines better long-term memory for whole faces that contain emotional information (Gupta and Srinivasan, 2009).

In recent years, researchers identified a relatively universal set of emotion expressions resumed in six main categories (happiness, sadness, fear, anger, surprise, and disgust) (Ekman, 2003).

Recognition of facial expressions depend both on accurate visuo-perceptual processes and on the correct categorization of the emotions. The right hemisphere plays a central role in processing emotional facial expressions (Gur et al., 2002). Emotional stimuli specifically activate a dominant right-lateralized process while automatic processing of emotional stimuli captures right hemispheric processing resources, transiently interfering with other right hemispheric functions (Hartikainen et al., 2000). Emotional distractors also produce transient interferences on the speed of visual search tasks in which stimuli are presented in the left visual hemifield (thus processed by the right hemisphere) (Gupta and Raymond, 2012). Furthermore, a significant slowing of letter targets presented in the left visual hemifield is detected when irrelevant distractors have high motivational salience regardless of their valence, an effect not found when targets appear in the right visual hemifield (Gupta et al., 2018). This indicates that the valence of irrelevant emotional distractors and the level of perceptual load in the task play an important role in the attentional capturing of distractors (Gupta et al., 2016).

Even if there is a functional overlap among the brain areas involved in recognition of different facial aspects (Calder and Young, 2005), neurophysiological data highlighted the central role of the amygdala and orbitofrontal circuits (Adolphs, 2002), the insula and striatum (Haxby et al., 2011) and the ventromedial prefrontal cortex (Mather, 2016) in the recognition of emotions.

Functional neurophysiological models suggest different structures involved in face identification and recognition of emotional expressions (Haxby et al., 2000; O’Toole et al., 2002). Fusiform face area (FFA) in the ventral temporal lobes are implicated in the perception of static facial features (Gobbini and Haxby, 2007; Vuilleumier and Pourtois, 2007), whereas the inferior occipital gyrus and the superior temporal sulcus (STS) are involved in processing the dynamic facial cues (Haxby et al., 2000; Adolphs, 2002; Ishai, 2008). The amygdala seems to be related especially to fear recognition. Fearful faces used as irrelevant stimuli can elicit an amygdala response only in the low perceptual load condition, suggesting that emotional faces capture attention despite their irrelevance in the task (Pessoa et al., 2005). The amygdala also shows sensitivity to the intensity of positive visual stimuli, as it is more activated in response to stimuli with higher intensity (Bonnet et al., 2015). The insula is related to disgust recognition, although intracranial electroencephalography (iEEG) highlights that amygdala and insula are more related to emotional relevance or biological importance of facial expressions than to specific emotions (Tippett et al., 2018).

Finally, many studies highlighted that basal ganglia and insula seem to be specifically involved in decoding facial expressions of disgust (Calder et al., 2001; Woolley et al., 2015). Insular activation is selectively reported to be active during processing of angry and, even more, of disgusted faces (Fusar-Poli et al., 2009). Amygdala is especially implicated in decoding facial expressions of fear (Fusar-Poli et al., 2009). A recent study (McFadyen et al., 2019) describes an afferent white matter pathway from the pulvinar to the amygdala that facilitates fear recognition. In this context, and in relation to old age, some authors (Ruffman et al., 2008) relate the relative sparing of some structures into the basal ganglia with increasing age to the lack of impairment of disgust recognition. Instead, the amygdala (related to fear recognition) could have a linear reduction in volume with age, although this is not as rapid as that occurring in some other brain areas such as the frontal lobes.

Brain lesions can affect the abilities in emotion recognition, inducing behavioral impairment (Hogeveen et al., 2016; Yassin et al., 2017), but also aging seems to play a considerable role in affecting recognition of facial expressions and consequently of emotions (Ruffman et al., 2008). Aging-related changes in emotional processing were found in the intra-network functional connectivity of the visual and sensorimotor networks implicated in internally directed cognitive activities (e.g., the Default Mode Network; DMN) and of the ECN (executive control network) involved in externally directed higher-order cognitive functions (Bressler and Menon, 2010; Uddin et al., 2010). A potential compensative role for aging-related decline in brain perceptual performance, as demonstrated by the progressive decline in visual and sensory domains with chronological aging was found for higher cognitive functions represented by ECN (Goh, 2011; Lyoo and Yoon, 2017).

Recent data (Olderbak et al., 2018) highlighted a peak of performance in emotion recognition in young people aged 15 to 30, with progressive decline after the age of 30. When compared to younger adults (18–30 years), older people (over 65 years) have more difficulties in correctly decoding negative emotions as anger, sadness, and fear (Ruffman et al., 2008; Grainger et al., 2015) showing a preservation for positive emotions such as happiness (Isaacowitz et al., 2007; Ruffman et al., 2008; Horning et al., 2012; West et al., 2012; Kessels et al., 2014). Furthermore, older people showed increased motivation to engage in prosocial behaviors in the context of relevant socio-emotional cues (Sze et al., 2012; Beadle et al., 2013).

There is also evidence that older people have difficulties in identifying emotions from dynamic bodily expressions (Ruffman et al., 2009), highlighting that dynamic cues may not be particularly beneficial in late adulthood (Grainger et al., 2015). Moreover, they are unable to detect when one emotion switches to another (Sullivan and Ruffman, 2004).

Eye-tracking studies highlighted that age-related differences in recognition of emotions could be explained by different face exploration patterns due to attentional impairment. Indeed, some studies observed that older people tend to fix on the mouth region, whereas young people spend attention on the eye areas (Wong et al., 2005; Sullivan et al., 2007; Murphy and Isaacowitz, 2010), which play a central role in decoding emotions such as anger, sadness and fear. Moreover, young people gaze more at the eye regions when stimuli are presented in a dynamic rather than still way. By contrast, older people do not show any difference between dynamic and still modality (Grainger et al., 2015).

Gender also seems to play a role in emotional regulation and in recognition of emotions but empirical studies of gender differences in emotion have produced far less consistent results. Neuroimaging data reports gender differences in the laterality of amygdala response, as it relates to subsequent memory (Cahill et al., 2001, 2004) and greater amygdala activity in men compared to women (Hamann et al., 2004; Schienle et al., 2005). As regards to emotional regulation, men and women show comparable amygdala response to negative images, but men display a greater decrease in amygdala activity during the use of a cognitive strategy for emotion regulation (reappraisal) and less activity in pre-frontal regions. In addition, women show greater ventral striatal activity during the down-regulation of negative emotion compared to men (McRae et al., 2008). Investigations about gender differences are conducted to understand the neural basis of complex social emotions. In this vein, distinct brain activations in response to sexual and emotional infidelity are detected in men and women. The former show greater activation in the amygdala and hypothalamus whereas the latter demonstrate greater activation in the posterior STS (Takahashi et al., 2006). Females seem to be more susceptible toward attention and evaluative bias for processing negative emotional information (Gupta, 2012). Females also show enhanced activity during the processing of negative information in potential event related components (N2 e P3b) (Li et al., 2008).

Unfortunately, little is known about differences in emotion perception abilities across lifespan separately for men and women, even if females show more ability from infancy (McClure, 2000). Recent research (Olderbak et al., 2018) conducted on a large community sample of persons ranging from younger than 15 to older than 60 years of age, highlighted less ability in males at all ages to read emotional faces. However, the gender differences in emotion recognition seem to decrease in magnitude with increasing age.

The aim of this study was to examine the role of age and gender effects on facial emotion recognition in relation to neuropsychological functions and face exploration strategies in a sample of young and old healthy people. We also aimed to explore the associations between emotion recognition and quality of life.

We expected a decreasing ability in emotion recognition correlated with the increasing of age, as well as differences between males and females. We also expected a negative correlation between cognitive functioning and face exploration patterns. Moreover we anticipate that subjective satisfaction of life might interfere with emotion recognition.

## Materials and Methods

### Participants

We enrolled a sample of 60 healthy people. They were healthy relatives of patients admitted to the neurological rehabilitation ward (where the study was conducted), and healthy controls from another sample (see Mancuso et al., 2015). Participants were divided into two groups: Younger Adults Group (YAG) aged from 28 to 54 and Older Adults Group (OAG) aged from 66 to 77.

The YAG was composed of 14 males, 14 females (Mean ± SD age 41.3 ± 9.34; range = 28–54). The OAG was composed of 16 males and 16 females (Mean ± SD age 70.7 ± 3.4; range = 66–77) (Table 1). There were no statistically significant differences between groups in education.

**TABLE 1:** Demographic details of the Younger and Older Adult Groups.

**Older adults group**					**Younger adults group**				
**No.**	**Gender**	**Age**	**Education**	**MMSE**	**No.**	**Gender**	**Age**	**Education**	**MMSE**
1	Female	74	10	28.86	1	Female	41	11	30
2	Female	70	17	28.85	2	Female	53	17	30
3	Female	71	11	27.86	3	Female	28	18	30
4	Female	68	8	30.53	4	Female	41	14	30
5	Female	71	8	29.2	5	Female	51	8	27
6	Female	74	17	28.85	6	Female	44	18	30
7	Female	67	5	31.27	7	Female	54	20	30
8	Female	73	5	31.03	8	Female	53	18	30
9	Female	72	15	28.85	9	Female	30	16	30
10	Female	67	8	29.53	10	Female	41	16	30
11	Female	73	5	31.3	11	Female	31	16	30
12	Female	75	5	31.03	12	Female	44	13	30
13	Female	69	15	28.46	13	Female	28	16	30
14	Female	72	5	32.03	14	Female	48	13	30
15	Female	68	15	28.46	15	Male	29	13	30
16	Female	71	16	28.86	16	Male	34	20	30
17	Male	76	8	30.2	17	Male	44	9	30
18	Male	68	10	29.49	18	Male	54	13	30
19	Male	69	8	29.53	19	Male	31	13	29
20	Male	75	13	28.86	20	Male	31	13	30
21	Male	69	8	28.47	21	Male	36	13	30
22	Male	77	8	30.2	22	Male	49	13	30
23	Male	66	13	27.49	23	Male	52	13	30
24	Male	66	8	29.53	24	Male	44	13	30
25	Male	66	18	30.2	25	Male	50	18	30
26	Male	67	13	29.49	26	Male	32	13	30
27	Male	77	15	28.85	27	Male	32	13	30
28	Male	74	10	29.86	28	Male	53	13	30
29	Male	68	13	29.49					
30	Male	71	15	28.86					
31	Male	66	11	29.49					
32	Male	75	10	28.86					
	Mean	70.7	10.8	29.5		Mean	41.3	14.4	29.8
	SD	3.4	4	1.05		SD	9.3	2.9	0.5

All people were screened for cognitive decline using the Mini-Mental State Examination (MMSE) (cut-off score of 24). Visual acuity was assessed in a functional way asking to people to correctly read a newspaper. The screen was approximately 35 cm from the eyes. The use of glasses or contact lenses was allowed. Exclusion criteria were a lower score than 24 on MMSE (Folstein et al., 1975) and the presence of a visual impairment uncorrected with glasses or contact lenses. All people enrolled in the study spoke Italian as their first language and none had positive history of previous neurological or psychiatric illness. Participants did not receive any remuneration for their participation. All subjects gave informed consent to the experimental procedure. The protocol was previously approved by the local Ethical Committee.

### Procedure

In order to detect differences in emotion recognition abilities, all participants underwent one session of about 2 h during which they were assessed for: emotion recognition, attention abilities, frontal functioning, memory functioning, and quality of life satisfaction.

During the execution of the emotion recognition test using the Pictures of Facial Affects (PoFA, Ekman and Friesen, 1976) and a modified version of PoFA (M-PoFA), all the eye movements of the subjects were recorded with the Tobii Eye Tracker in order to detect the exploration modality adopted by each participant.

#### Test for Emotion Recognition

Pictures of Facial Affects (Ekman and Friesen, 1976) consists of 110 photographs of human faces representing the six basic emotions (happiness, sadness, fear, anger, surprise, and disgust), plus one neutral expression.

We tested the recognition abilities in two different ways. In the first (Single PoFA Recognition) we displayed the 110 single photos for 5 s in a random sequence on the screen connected to an eye tracker in order to record all the eye movements made by subjects during image exploration. After each photo a list with the names of the seven emotions (the six from Ekman and the neutral one) appeared on the screen. The participants were asked to match the just-observed emotion with one of the seven choices indicating: happiness, sadness, fear, anger, surprise, disgust and neutral expression. The aim of this test was to evaluate the ability of people to correctly recognize the emotions. The number of correct match between the observed emotion on the photo and the name of the emotion were recorded obtaining seven scores (one for each emotion) and a total score (number of correct emotions identified).

In the second way (Matching of Pictures of Facial Affects: M-PoFA), we displayed on the screen the same photographs of PoFA in pairs for 5 s, in a randomized order. The subjects had to decide whether the emotions represented in the two faces were the same or different. The aim of this test was to evaluate the possible loss of the pattern which mediates the recognition of emotions, as opposed to the ability to correctly recognize and name the emotion. Three scores were detected: M-PoFA total correct score (number of correct same or different couples identified), M-PoFA total score for identical emotion pair (number of correct identical emotion pairs identified), M-PoFA total score for not identical emotion pairs (number of correct not identical emotion pairs identified).

Eye movements recording: Eye movements were recorded with the Tobii Eye Tracker (My Tobii 1750; Technology AB, Danderyd, Sweden) using infrared diodes to generate reflection patterns on the corneas of the user’s eyes, collected by image sensors. The eyes and the corneal reflection patterns were processed by image algorithms to calculate the three-dimensional position of eyeball and gaze point of viewing on the screen. Before each task, a calibration procedure was carried out.

Eye movements were recorded for the whole time spent by people to execute the PoFA test in both modalities. The registration was carried out only during photos presentation in order to detect eye movements in face stimuli scanning. Each picture displayed was divided into different areas of interest (AOI), created by means of an AOI tool button that allows to define an AOI on a static image by choosing a desired shape tool. The AOIs created on each picture were: right eye AOI (od-AOI), left eye AOI (os-AOI), frontal AOI, mouth AOI, distractor-fixation AOI (the distractor was an irrelevant small number in the upper left of each photo), global AOI (the whole photo area) and area of no interest (Not on AOI - the area of the computer screen outside the photo) with a clear net.

For each of these AOI, we measured the following parameters: time to first fixation (the time from the start of the stimulus display until the subject fixates an AOI for the first time, measured in seconds); fixation length (duration of each individual fixation within an AOI, in seconds); first fixation duration (duration of the first fixation on an AOI, in seconds); fixation count (number of times the participant fixates on an AOI); observation length (duration of all visits within each AOI, in seconds); observation count (number of visits within each AOI); fixation before (number of times the participant fixates on the stimulus before fixating on an AOI for the first time) and participants% (percentage of recordings in which participants fixated at least once within an AOI).

#### Test for Attentional Abilities

Sustained Attention to Response Task (SART, Robertson et al., 1997): this test was devised to evaluate sustained attention and comprises the numbers 1–9 appearing 225 times in random order and in different sizes in a white font on a black computer screen. Subjects had to respond to the appearance of each number by pressing a button, except when the number was a 3, which occurred 25 times in total. The following measures of response accuracy were assessed: mean reaction time in milliseconds, calculated over correct response trials, number of commission errors (key presses when no key should have been pressed) and number of omission errors (absent presses when a key should have been pressed).

Trail Making Test (TMT, Giovagnoli et al., 1996): composed of two subtests: part A measures the speed of visual search and psychomotor ability by connecting numbers from 1 to 25 typed on a sheet; part B measures the ability to alternate between two sequences of numbers and letters. Every subtest is preceded by a run-in, whose correct execution represents the condition to proceed with the administration of the test. Scores are given according to the time needed to complete subtests A and B. Subtraction of part B from part A gives an estimation of attentional switching capacity.

#### Test for Frontal Functioning

Frontal Assessment Battery (FAB, Apollonio et al., 2005): a battery for general screening of executive functions divided into six subtests. Each of them assesses an aspect of executive functions: conceptualization of similarities, lexical fluency, motor programming, response to conflicting instructions, go-no go task, prehension behavior. The score for every subtests ranges from 0 to 3. The maximum score is 18, which is corrected for age and schooling according to standardization norms.

#### Test for Memory

Corsi Supra-Span Learning (CSSL, Capitani et al., 1991): a supra-span sequence of 8-blocks is showed to the patient, who is asked to reproduce it immediately after its presentation. The examiner presents the same sequence until the patient reproduces it correctly three times consecutively, up to a maximum of 18 trials. If the subject does not succeed within 18 trials, the task is interrupted. Each sequence given by patients is scored on the basis of the probability of giving the correct response (or fragments of it) by chance. Subjects that reached the criterion before the 18th trial received a score corresponding to the correct performance for the remaining trials up to the 18th. The maximum score of the CSSL is 29.16, which is corrected for age and schooling according to standardization norms.

#### Test Assessing Satisfaction of Life

Satisfaction Profile (SAT-P, Majani and Callegari, 1998): self-administered questionnaire of 32 items assessing areas that reflects subjective satisfaction of life: sleep, eating, physical activity, sexual life, emotional status, self-efficacy, cognitive functioning, work, leisure, social and family relationships, and financial situation. Patients were requested to evaluate their personal satisfaction in the last month for each of the 32 items on a 10 cm horizontal scale ranging from “extremely dissatisfied” to “extremely satisfied.” It provides two kinds of scores: the analytic scoring that consists of 32 scores (one for each item) and the factorial scoring that consists of 5 scores, one for each of the factor extracted by factor analysis: psychological functioning, physical functioning, work, sleep/eating/leisure, and social functioning. In SAT-P higher scores correspond to better satisfaction of life (range 0–100 for both analytic and factorial scoring).

### Statistical Analysis

In order to test the influence of age and gender on PoFA and M-PoFA scores, we used Multivariate Analysis of Variance (MANOVA) where assumptions for collinearity were met, otherwise univariate test were performed. *Post hoc* comparisons were carried out by means of Bonferroni test (*p* < 0.05).

Analysis of covariance (ANCOVA) was used to compare groups on the emotion recognition measures in order to control the bias of possible confounding variables. Thus, variables emerged as significant in the MANOVAs in affecting emotion recognition as confounders, were entered into the analysis as covariates.

Age effects on SAT-P were detected comparing younger and older adults by means of non-parametric Mann-Whitney test for independent samples. Spearman ρ correlation coefficient for categorical variables was used to correlate satisfaction of life with emotion recognition.

All tests were two tailed and considered significant if *p* < 0.05.

The Statistical Package for Social Science, version 20.0 (SPSS Inc., Chicago, IL, United States), was used for statistical analyses.

## Results

Table 2 shows Mean and Standard Deviation (SD) for PoFA single emotion recognition scores, PoFA total correct emotion recognition scores, summed over the seven emotions (total = 110) and for M-PoFA total correct score, M-PoFA total score for identical emotion pair, M-PoFA total score for not identical emotion pair.

**TABLE 2:** Number of emotion items recognized correctly (SD) for both age groups and both genders.

	**Younger adults group**			**Older adults group**		
	**Total**	**Men**	**Women**	**Total**	**Men**	**Women**
**PoFA**						
Happiness	17.36 (0.78)	17.29 (0.83)	17.43 (0.76)	16.91 (1.42)	17.06 (1.39)	16.75 (1.48)
Sadness	14.89 (2.13)	14.43 (2.14)	15.36 (2.10)	13.22 (2.77)	12.75 (2.62)	13.69 (2.91)
Fear	11.61 (3.46)	10.64 (3.27)	12.57 (3.48)	7.94 (3.81)	8.13 (3.12)	7.75 (4.49)
Anger	15.25 (2.24)	14.57 (2.82)	15.93 (1.21)	14.03 (2.57)	13.00 (3.03)	15.06 (1.48)
Surprise	13.75 (0.52)	13.79 (0.43)	13.71 (0.61)	13.19 (1.26)	13.38 (0.72)	13.00 (1.63)
Disgust	13.89 (1.83)	13.86 (1.56)	13.93 (2.13)	13.72 (1.82)	13.13 (1.93)	14.31 (1.54)
Neutral	13.21 (1.34)	13.36 (1.15)	13.07 (1.54)	12.75 (2.34)	12.25 (3.13)	13.25 (1.00)
Total Correct	99.96 (8.27)	97.93 (8.33)	102.00 (7.98)	91.75 (8.54)	89.69 (7.31)	93.81 (9.40)
**M-PoFA**						
Total Correct	118.57 (5.79)	117.64 (4.60)	119.50 (6.82)	113.91 (7.77)	110.63 (8.80)	117.19 (4.93)
Identical Pair	62.43 (2.75)	61.57 (3.23)	63.29 (1.94)	59.88 (4.65)	57.50 (5.28)	62.25 (2.18)
Not Identical Pair	56.14 (4.34)	56.07 (2.95)	56.21 (5.51)	54.03 (4.43)	53.13 (4.57)	54.94 (4.23)

*Maximum for each emotion category in PoFA, Happiness = 18; Sadness, 17; Fear, 15; Anger, 17; Surprise, 14; Disgust, 15; Neutral, 14; Total Correct, 110. Maximum for each M-PoFA score: Total Correct, 126; Identical Pair, 66; Not Identical Pair, 60.*

### Effects of Age on Emotion Recognition in the Sample Divided in Younger and Older Adults Group

Comparisons between YAG and OAG on PoFA and on M-PoFA were carried out by means of a multivariate ANOVA. We used the “group” variable as the between group factor and PoFA scores (happiness, sadness, fear, anger, surprise, disgust, neutral) and M-PoFA scores (total score, total score for identical emotion pair, total score for not identical emotion pairs) as the dependent variables. There was a significant effect of the group factor on emotion recognition variables (Wilk’s Lambda = 0.65, *F*_[__9_,_50__]_ = 2.98; *p* = 0.006; η^2^ = 0.35). The YAG demonstrated significantly greater scores in sadness (*F*_[__1_,_58__]_ = 6.7; *p* = 0.012; η^2^ = 0.10), fear (*F*_[__1_,_58__]_ = 15.09; *p* = 0.000; η^2^ = 0.20), surprise recognition (*F*_[__1_,_58__]_ = 4.88; *p* = 0.031; η^2^ = 0.07), PoFA total score (*F*_[__1_,_58__]_ = 14.2; *p* = 0.000; η^2^ = 0.19), M-PoFA total correct score (*F*_[__1_,_58__]_ = 14.2; *p* = 0.000; η^2^ = 0.19) and M-PoFA total score for identical emotion pair (*F*_[__1_,_58__]_ = 6.45; *p* = 0.014; η^2^ = 0.10) when compared to OAG.

The Supplementary Table S1 shows the comparison between younger and older adults on neuropsychological variables (SART, TMT, FAB, CSSL) and on eye movements variables. A MANOVA was conducted using the between group factor “group” as the independent variable and neuropsychological variables as dependent measures (SART, TMT, FAB, CSSL). A significant effect of the group factor on cognitive functioning variables was detected (Wilk’s Lambda = 0.58, *F*_[__7_,_52__]_ = 5.3; *p* = 0.000; η^2^ = 0.41). The younger adults showed significant faster reaction time (*F*_[__1_,_58__]_ = 24.1; *p* = 0.000; η^2^ = 0.29), fewer omission errors (*F*_[__1_,_58__]_ = 5.78; *p* = 0.019; η^2^ = 0.09) and commission errors (*F*_[__1_,_58__]_ = 7.92; *p* = 0.007; η^2^ = 0.12) in the SART test, better attentional shifting abilities in TMT B-A (F_[__1_,_58__]_ = 11.4; *p* = 0.001; η^2^ = 0.16) and higher long term visuospatial memory scores on CSSL (*F*_[__1_,_58__]_ = 13.01; *p* = 0.001; η^2^ = 0.18) compared to OAG.

As regards comparisons between YAG and OAG on eye movements pattern, univariate ANOVAs showed that older adults exhibited lower duration of each individual fixation not on AOI (Not on AOI Fixation Length – *F*_[__1_,_58__]_ = 11.3; *p* = 0.001; η^2^ = 0.16), shorter duration of the first fixation not on an AOI (not on AOI first fixation duration – *F*_[__1_,_58__]_ = 3.99; *p* = 0.05; η^2^ = 0.06) compared to the younger adults. The younger adults showed fewer numbers of times in which each participant fixates the distractor (AOI distractor Fixation Count – *F*_[__1_,_58__]_ = 4.24; *p* = 0.044; η^2^ = 0.07), lower duration of all visits into frontal AOI (AOI frontal Observation Length – *F*_[__1_,_58__]_ = 3.9; *p* = 0.05; η^2^ = 0.06) and into distractor AOI (AOI distractor Observation Length – *F*_[__1_,_58__]_ = 6.14; *p* = 0.016; η^2^ = 0.09) compared to the older adults.

Regarding single emotions domains, *F* did not reach significance after including SART, TMT B-A, CSSL, and eye-gaze fixations characteristics (Not on AOI Fixation Length, Not on AOI first fixation duration, AOI distractor Fixation Count, AOI frontal Observation Length and AOI distractor Observation Length) as covariates in happiness, sadness, anger, surprise, disgust, neutral emotion recognition and PoFA total score. Also for M-PoFA, *F* did not reach significance after inclusion of covariates SART, TMT B-A, CSSL, and eye-gaze fixations characteristics (Not on AOI Fixation Length, Not on AOI first fixation duration, AOI distractor Fixation Count, AOI frontal Observation Length and AOI distractor Observation Length).

When SART, TMT B-A, CSSL, and eye-gaze fixations characteristics (Not on AOI Fixation Length, Not on AOI first fixation duration, AOI distractor Fixation Count, AOI frontal Observation Length and AOI distractor Observation Length) were included as covariates, the only significant difference between the younger and the older adults was found in fear recognition (*F*_[__1_,_49__]_ = 4.37; *p* = 0.042; η^2^ = 0.08). ANCOVA analysis revealed statistically significant differences in fear recognition between young and old adults when adjusted for cognitive functioning and eye-gaze fixations characteristics: adjusted means of fear recognition were significantly higher in the younger group than in the older group (corrected) (*p* = 0.04) (Table 3).

**TABLE 3:** Comparison between Younger and Older Adult Groups in emotion recognition variables using neuropsychological variables and eye movement variables as confounding covariates.

	**Younger adults group**	**Older adults group**	**ANCOVA**	
**Emotion recognition**	**Mean (SE)**	**Mean (SE)**	***F***	***p* value**
**PoFA**				
Happiness	17.02 (0.26)	17.2 (0.24)	0.18	>0.05
Sadness	14.3 (0.57)	13.6 (0.52)	0.66	>0.05
Fear	11.2 (0.91)	8.2 (0.83)	4.4	0.04^∗^
Anger	15.1 (0.55)	14.1 (0.5)	1.3	>0.05
Surprise	13.43 (0.2)	13.46 (0.18)	0.006	>0.05
Disgust	13.2 (0.41)	14.2 (0.38)	2.2	>0.05
Neutral	12.7 (0.39)	13.1 (0.36)	0.4	>0.05
**M-PoFA**				
Total correct	97.2 (1.8)	94.09 (1.7)	1.16	>0.05
Identical pair	60.7 (0.78)	61.3 (0.71)	0.28	>0.05
Not identical pair	55.4 (1.03)	54.6 (0.94)	0.21	>0.05

*Asterisks indicate statistically significant differences at an alpha of 0.05.*

### Effects of Gender on Emotion Recognition in Younger and Older Adults

Multivariate ANOVAs were conducted in the older and, separately, in the younger adults. In each group we conducted two separate MANOVAs in order to compare males and females on emotion recognition and cognitive functioning. To test the influence of gender on cognitive functioning we used gender as independent variable and neuropsychological variables (SART, TMT, FAB, CSSL) as dependent measures. To test exploration strategies, males and females were compared by means of univariate ANOVAs entering as dependent variable each Eye tracker parameter. As regards emotion recognition, in the MANOVA we used gender as the independent variable and PoFA scores (happiness, sadness, fear, anger, surprise, disgust, neutral) and M-PoFA scores (total score, total score for identical emotion pair, total score for not identical emotion pairs) as dependent variables.

In the YAG, no significant differences between males and females were detected in emotion recognition (Wilk’s Lambda = 0.7, *F*_[__9_,_18__]_ = 0.83; *p* = 0.59; η^2^ = 0.29), in cognitive functioning (Wilk’s Lambda = 0.78, *F*_[__6_,_21__]_ = 0.9; *p* = 0.46; η^2^ = 0.21) and in eye-gaze fixations characteristics (all *p* > 0.05).

In the OAG, although the MANOVA didn’t show a significant effect of gender on emotion recognition variables (Wilk’s Lambda = 0.65, *F*_[__9_,_22__]_ = 1.3; *p* = 0.28; η^2^ = 0.34), between-subjects effects showed a significant effect of gender on anger recognition (*F*_[__1_,_30__]_ = 5.9; *p* = 0.02; η^2^ = 0.16), on M-PoFA total score for identical emotion pair (*F*_[__1_,_30__]_ = 11.07; *p* = 0.002; η^2^ = 0.27) and on M-PoFA total correct score (*F*_[__1_,_30__]_ = 6.7; *p* = 0.014; η^2^ = 0.18). Old males showed significant lower scores with respect to old females in anger recognition (Mean = 13; SE = 0.59 vs. Mean = 15.06; SE = 0.59), in M-PoFA total correct score (Mean = 110.6; SE = 1.7 vs. Mean = 117.1; SE = 1.7) and in M-PoFA total score for identical emotion pair (Mean = 57.5; SE = 1 vs. Mean = 62.2; SE = 1).

No significant effect of gender was found on cognitive functioning in the older adults (Wilk’s Lambda = 0.64, *F*_[__9_,_22__]_ = 1.3; *p* = 0.27; η^2^ = 0.35).

In the older adults, eye-gaze fixations characteristics on which the males and females differed were: AOI-od-Time-to-first-fixation (*F*_[__1_,_30__]_ = 3.88; *p* = 0.058; η^2^ = 0.11) (Mean females = 1812; SE = 178.6 vs. Mean males = 2310; SE = 178.6), AOI-os-First-fixation-duration (*F*_[__1_,_30__]_ = 5.38; *p* = 0.02; η^2^ = 0.15) (Mean females = 294.8; SE = 19.9 vs. Mean males = 360.5; SE = 19.9), AOI-od-First-fixation-duration (*F*_[__1_,_30__]_ = 4.14; *p* = 0.05; η^2^ = 0.12) (Mean females = 284.1; SE = 15.01 vs. Mean males = 327.4; SE = 15.01), AOI-frontal-Observation-Count (*F*_[__1_,_30__]_ = 4.2; *p* = 0.04; η^2^ = 0.12) (Mean females = 3748; SE = 269.7 vs. Mean males = 2960; SE = 269.7), AOI-frontal-Fixations-Before (*F*_[__1_,_30__]_ = 4.36; *p* = 0.04; η^2^ = 0.12) (Mean females = 8788.6; SE = 986 vs. Mean males = 11703; SE = 986) and AOI-od-Participant-perc (*F*_[__1_,_30__]_ = 3.88; *p* = 0.05; η^2^ = 0.11) (Mean females = 1812.06; SE = 178.6 vs. Mean males = 2310; SE = 178.6).

Old males spent more time to reach fixation on the right eye AOI for the first time (AOI-od-Time-to-first-fixation), showed more duration of the first fixation on the right eye and on the left eye AOI (AOI-os-First-fixation-duration and AOI-od-First-fixation-duration), a higher number of times in which they fixated on the stimulus before fixating on frontal AOI for the first time (AOI-frontal-Fixations-Before) and more recordings in which they fixated at least once into the right eye AOI (expressed as a fraction of the total number of recordings) (AOI-od-Participant-perc) with respect to old females.

Old females made a significantly higher number of visits (i.e., the interval of time between the first fixation on the AOI and the next fixation outside the AOI) into frontal AOI (AOI-frontal-Observation-Count) with respect to males (Table 4).

**TABLE 4:** Comparison between males and females in the Older Adults Group on neuropsychological variables and on eye movement variables.

	**Older adults group**		**ANOVA**	
	**Males**	**Females**		
**Eye movements recording**	**Mean (SE)**	**Mean (SE)**	***F***	***p* value**
AOI-od-Time to-first-fixation	2310 (178.6)	1812 (178.6)	3.88	0.05^∗^
AOI-os First-fixation-duration	360.5 (19.9)	294.8 (19.9)	5.38	0.02^∗^
AOI-od-First-fixation-duration	327.4 (15.01)	284.1 (15.01)	4.14	0.05^∗^
AOI-frontal-Observation-Count	2960 (269.7)	3748 (269.7)	4.2	0.04^∗^
AOI-frontal-Fixations-Before	11703 (986)	8788.6 (986)	4.36	0.04^∗^
AOI-od-Participant-perc	2310 (178.6)	1812.06 (178.6)	3.88	0.05^∗^

*We reported only variables that reached statistical significance. Asterisks indicate statistically significant differences at an alpha of 0.05.*

With regards to emotion recognition, in the older adults *F* did not reach significance after including eye-gaze fixations characteristics (AOI-od-Time-to-first-fixation, AOI-os-First-fixation-duration, AOI-od-First-fixation-duration, AOI-frontal-Observation-Count, AOI-frontal-Fixations-Before, AOI-od-Participant-perc) as covariates in happiness, sadness, anger, surprise, disgust, neutral expression recognition, and PoFA total score.

Differences between males and females in the older adults in anger recognition can be attributed to face exploration modality: AOI-od-First-fixation-duration exerts a significant effect on anger recognition (*F*_[__1_,_25__]_ = 9.17; *p* = 0.006; η^2^ = 0.26).

After inclusion of covariates there was a significant difference in the older adults between males and females only in M-PoFA total score for identical emotion pair (*F*_[__1_,_25__]_ = 6.9; *p* = 0.014; η^2^ = 0.21). ANCOVA analysis revealed that recognition of identical pairs was significantly higher in females than in males (adjusted Mean = 61.8; SE = 0.98 vs. adjusted Mean = 57.8; SE = 0.98) (Table 5).

**TABLE 5:** Comparison between males and females in the Younger and the Older Adult Groups in emotion recognition variables using neuropsychological variables and eye movements variables as confounding covariates.

	**Younger Adults Group**		**ANCOVA**		**Older Adult Group**		**ANCOVA**	
	**Males**	**Females**			**Males**	**Females**		
**Emotion recognition**	**Mean (SE)**	**Mean (SE)**	***F***	***p* value**	**Mean (SE)**	**Mean (SE)**	***F***	***p* value**
**PoFA**								
Happiness	17.1 (0.24)	17.5 (0.24)	0.72	>0.05	17.08 (0.4)	16.7 (0.4)	0.3	>0.05
Sadness	14.5 (0.55)	15.2 (0.55)	0.58	>0.05	12.9 (0.84)	13.4 (0.84)	0.1	>0.05
Fear	10.2 (0.87)	12.9 (0.87)	4.1	>0.05	8.5 (1.1)	7.3 (1.1)	0.4	>0.05
Anger	14.5 (0.51)	15.9 (0.51)	3.3	>0.05	13.5 (0.59)	14.4 (0.59)	0.9	>0.05
Surprise	13.74 (0.14)	13.75 (0.14)	0.001	>0.05	13.5 (0.33)	12.8 (0.33)	1.5	>0.05
Disgust	13.7 (0.48)	14.02 (0.48)	0.12	>0.05	13.3 (0.49)	14.08 (0.49)	0.9	>0.05
Neutral	13.1 (0.24)	13.3 (0.24)	0.26	>0.05	12.6 (0.66)	12.8 (0.66)	0.05	>0.05
**M-PoFA**								
Total correct	117 (1.2)	120.1 (1.2)	2.5	>0.05	111.8 (1.7)	115.9 (1.7)	2.1	>0.05
Identical pair	61.4 (0.72)	63.3 (0.72)	3.01	>0.05	57.8 (0.98)	61.8 (0.98)	6.9	0.01^∗^
Not identical pair	55.5 (0.98)	56.7 (0.98)	0.68	>0.05	54 (1.2)	54.06 (1.2)	0.001	>0.05

*Asterisks indicate statistically significant differences at an alpha of 0.05.*

### Relations Between Satisfaction Profile and Emotion Recognition

Factor 3 (Work) (*Z* = −2.5, *p* = 0.02) and factor 4 (*Z* = −2.5, *p* = 0.01) (Sleep/feeding/free time) scores of SAT-P were significantly higher in the older adults than in the younger adults.

Bivariate correlations between SAT-P and emotion recognition in the total sample, in the YAG and in the OAG are documented in Table 6. A negative correlation emerged between factor 3 (Work) and anger recognition (ρ = −0.25, *p* = 0.05). Factor 4 of SAT-P (Sleep/feeding/free time) was negatively correlated with fear (ρ = −0.24, *p* = 0.05), anger (ρ = −0.28, *p* = 0.02), surprise recognition (ρ = −0.25, *p* = 0.05), PoFA total score (ρ = −0.3, *p* = 0.005) and M-PoFA total score for not identical emotion pair (ρ = −0.3, *p* = 0.007).

**TABLE 6:** Correlations between SAT-P and emotion recognition in the total sample, in the YAG and in the OAG.

	**PoFA**																**M-PoFA**			
**SAT-P factors**	**Happiness**		**Sadness**		**Fear**		**Anger**		**Surprise**		**Disgust**		**Neutral**		**Total correct score**		**Identical pair**		**Different pair**	
	**ρ**	***p***	**ρ**	***p***	**ρ**	***p***	**ρ**	***p***	**ρ**	***p***	**ρ**	***p***	**ρ**	***p***	**ρ**	***p***	**ρ**	***p***	**ρ**	***p***
**Total sample**																				
Psychological functioning	0.04	0.7	–0.09	0.4	–0.1	0.4	–0.2	0.1	–0.04	0.7	–0.07	0.5	–0.02	0.8	–0.1	0.1	–0.07	0.5	–0.2	0.1
Physical functioning	0.12	0.3	–0.2	0.04	–0.08	0.4	–0.1	0.1	–0.03	0.7	–0.2	0.06	–0.02	0.8	–0.2	0.09	–0.05	0.6	–0.2	0.1
Work	0.16	0.2	–0.17	0.1	–0.03	0.7	−**0.25**	**0.05^∗^**	–0.006	0.9	–0.08	0.5	–0.1	0.2	–0.1	0.2	–0.13	0.3	–0.2	0.06
Sleep/feeding/free time	–0.05	0.7	–0.22	0.08	−**0.24**	**0.05**^∗^	−**0.28**	**0.02**^∗^	−**0.25**	**0.05**^∗^	–0.1	0.1	–0.1	0.2	−**0.3**	**0.005^∗^**	–0.1	0.2	−**0.3**	**0.007^∗^**
Social functioning	0.17	0.18	–0.17	0.19	–0.06	0.6	–0.02	0.8	–0.05	0.6	–0.07	0.5	–0.1	0.2	–0.1	0.4	–0.04	0.7	–0.2	0.1
**Younger adults group**																				
Psychological functioning	0.14	0.45	–0.3	0.1	–0.18	0.33	–0.3	0.11	0.009	0.96	–0.19	0.33	–0.08	0.65	–0.19	0.31	–0.002	0.99	–0.22	0.24
Physical functioning	0.22	0.25	−**0.45**	**0.01^∗^**	–0.05	0.79	–0.19	0.33	0.005	0.98	−**0.45**	**0.01^∗^**	0.07	0.7	–0.25	0.19	0.13	0.49	–0.19	0.32
Work	0.12	0.53	–0.32	0.09	–0.08	0.68	–0.25	0.18	–0.14	0.45	–0.08	0.65	–0.17	0.37	–0.11	0.57	–0.002	0.99	–0.19	0.31
Sleep/feeding/free time	0.12	0.53	–0.3	0.11	–0.25	0.2	–0.21	0.27	–0.12	0.52	−**0.39**	**0.03^∗^**	–0.29	0.12	–0.24	0.2	–0.005	0.98	–0.13	0.5
Social functioning	0.23	0.22	–0.23	0.22	–0.06	0.75	0.02	0.88	0.009	0.96	–0.1	0.59	0.01	0.93	–0.09	0.63	0.08	0.66	–0.23	0.22
**Older adults group**																				
Psychological functioning	–0.006	0.97	0.11	0.55	0.07	0.67	–0.07	0.69	–0.06	0.72	0.08	0.63	0.03	0.84	–0.11	0.52	–0.07	0.67	–0.12	0.48
Physical functioning	0.06	0.7	–0.2	0.89	0.02	0.87	–0.09	0.61	0.02	0.87	–0.06	0.72	0.004	0.98	–0.09	0.6	–0.13	0.47	–0.16	0.35
Work	0.29	0.11	0.04	0.8	0.34	0.05	–0.08	0.65	0.24	0.18	–0.01	0.93	–0.03	0.86	0.19	0.28	–0.05	0.77	–0.17	0.34
Sleep/feeding/free time	–0.13	0.45	–0.1	0.55	0.07	0.67	–0.1	0.58	–0.22	0.22	0.07	0.7	0.63	0.73	–0.18	0.3	–0.1	0.58	−**0.37**	**0.03^∗^**
Social functioning	0.16	0.35	–0.05	0.75	0.1	0.58	0.06	0.72	–0.003	0.98	–0.004	0.98	–0.26	0.15	–0.03	0.85	0.03	0.86	–0.09	0.59

*ρ: Spearman’s correlation coefficient. Significant *p* values are shown in bold exponents. Satisfaction profile (SAT-P): Factor 1, psychological functioning; Factor 2, physical functioning; Factor 3, work; Factor 4, sleep/feeding/free time; Factor 5, social functioning. Asterisks indicate statistically significant differences at an alpha of 0.05.*

As shown in Table 6, in the YAG the factor 2 (Physical functioning) was significantly negatively correlated with sadness (ρ = −0.45, *p* = 0.01) and disgust (ρ = −0.45, *p* = 0.01) recognition; factor 4 (Sleep/feeding/free time) was significantly negatively correlated with disgust recognition (ρ = −0.39, *p* = 0.03).

For the older adults, bivariate correlations showed a significant negative correlation between factor 4 (Sleep/feeding/free time) and recognition of not identical emotion pairs (ρ = −0.37, *p* = 0.03).

## Discussion

The emotion recognition of a sample (*n* = 60) of individuals (30 males, 30 females) with different ages was examined. The aim of the study was to assess the role of age and gender effects on facial emotion recognition in relation to neuropsychological functions and face exploration strategies in a sample of young and old healthy people.

### Differences in Emotion Recognition: The Influence of Aging

Many previous studies highlighted that older adults perform worse than younger adults in perceiving emotions, particularly when recognizing anger, sadness, and fear (Isaacowitz and Stanley, 2011; Sullivan et al., 2017), whereas a recent study underlined that age differences in emotion perception were limited to very low intensity expressions (Mienaltowski et al., 2019).

In our study, the YAG demonstrated significantly better scores on sadness, fear, surprise recognition and on PoFA total score compared to the OAG. In addition, the older adults got lower scores on matching identical Pictures of Facial Affects in M-PoFA, presenting more difficulties than younger adults in matching identical pairs of emotions, suggesting that they lost the ability to recognize the similarities or the differences between two specific emotional patterns, regardless of their meaning.

Our data was in contrast with previous works that reported age differences in two-choice matching tasks in which emotion recognition requires to simply perceive similarities in stimuli features only when stimuli display less intense emotions (Orgeta, 2010; Mienaltowski et al., 2013). Our paradigm required a perceptual judgment in M-PoFA test, thus eliminating cognitive factors associated with multiple verbal labels of emotion to choose from, but the facial expressions that we used in M-PoFA, as derived from PoFA test, represent highly intense emotional expressions (Carroll and Russell, 1997).

Furthermore, according to our data, age seems to negatively affect emotion recognition, but there are no differences in anger (in contrast with Sullivan et al., 2017), happiness, and disgust recognition. The absence of age differences in anger and happiness recognition in our sample is in accordance with the results from Mienaltowski et al. (2019) and it might be correlated to the high intensity of emotions expressed in the stimuli. The absence of differences reported in disgust may also be correlated to a more “visceral” emotion that involves social cognitive mechanisms to regulate or anticipate and avoid exposure to pathogens and toxins (Kavaliers et al., 2018), necessary to preserve physical integrity. Disgust is also an emotion linking inner somatic visceral perceptions to external stimuli. Perhaps, we could explain the preservation of disgust recognition in the older adults as a special type of attention given to the body, both because it is frailer and because in older age psychological distress is also expressed through body and somatic symptoms.

In addition, a meta-analytic review (Ruffman et al., 2008) observed a decline in older adults of emotion recognition with the exception of disgust (as in our study), with a non-significant trend for older adults to be better than younger adults in disgust recognition. Moreover, some authors (Ebner et al., 2011) observed an age-group related difference in misattribution of expressions, in which younger adults were more likely to label disgusted faces as angry, whereas older adults were more likely to label angry faces as disgusted.

Considering the SAT-P our results, although to be considered with caution, showed a tendency toward more satisfaction with some life dimensions in older adults than in younger subjects, especially with regard to sleep/feeding/free time, activities and economic factors. Moreover, in our sample, both in the older adults and in the younger subjects, satisfaction of life is negatively correlated with recognition of essentially negative emotions, such as sadness and disgust in the YAG, with anger, fear and surprise seen across the total sample. Previous studies have underlined a mood-congruent effect in processing face emotions. Voelkle et al. (2014) observed that older adults were more likely to perceive happiness in faces when in a positive mood and less likely to do so when in a negative mood, while a recent study (Lawrie et al., 2018) demonstrated how emotion perception can change when a controlled mood induction procedure is applied to alter mood in young and older subjects. Moreover, some authors (Ruffman et al., 2008) also observed that older adults present a positive bias in attention and memory for positive emotional information compared to negative information, as an adaptive strategy to maintain emotion regulation and avoid social conflict. In this sense, an Eye-tracking study (Wirth et al., 2017) observed that older adults, when compared to younger adults, showed fixation patterns away from negative image content, while they reacted with greater negative emotions.

However, of the six basic emotions, only happiness is unambiguously a positive emotion. Indeed, in our sample, older adult subjects did not have difficulties in happiness recognition when compared to YAG.

Moreover, in our sample, the difficulty in recognizing patterns of emotions that are useful in discriminating different pairs of emotions also seems to be related to life satisfaction. Indeed, both in the general sample and particularly in the older adults, we find an inverse correlation between accurate comparisons of different pairs of emotions on the one hand, and some aspects of life satisfaction on the other (sleep/feeding/free time).

Considering cognitive functions, as expected, the YAG was significantly faster and more accurate in sustained attention, showing better attentional shifting abilities in TMT B-A and higher long-term visuospatial memory scores on CSSL compared to the older adults.

In our study, the older adults explored the tops of faces more than younger adults. However, a previous study (Sullivan et al., 2017) underlines that time spent by older men looking at mouths, alongside time spent by women looking at the eyes, correlated with the emotion recognition of both genders respectively. As a result, looking more at the forehead is probably a disadvantage for older males.

After covarying (ANCOVA) for cognitive function (sustained attention test, attentional shifting abilities, long term visuospatial memory abilities) and exploration strategies, the older adults showed significantly impaired abilities only with fear recognition. The older adults showed similar abilities with happiness, sadness, anger, surprise, disgust, neutral expression recognition and PoFA total score when compared to the younger adults. In accordance with a recent study (Gonçalves et al., 2018), although older adults exhibited a worse performance in neuropsychological tests with respect to the younger group, this disadvantage seemed not to be reflected in emotion identification, thus suggesting that in our study neuropsychological functioning did not influence the emotion identification ability.

Also, other studies pointed out that in aging there are both attentional differences and perceptual deficits contributing to less accurate emotion recognition (Birmingham et al., 2018) and that facial emotion perception is related to the ability of perceiving faces (face perception), remembering faces (face memory), remembering faces with emotional expressions (emotion memory), and general mental ability (Hildebrandt et al., 2015).

From our data, the impairment of fear recognition seems to be unrelated both to cognitive functions and to exploration strategies in older adults. Therefore, in our sample, the difference in emotions processing between younger and older adults seems to be related to cognitive functions and strategy of face exploration for all emotions, except for fear recognition. Fear seems to be unrelated to these components, as it is a “phylogenetically ancient” emotion, closely related to the survival of the species. Indeed, fear emotion can be processed quickly and automatically by deep brain structures (amygdala), without involving the cortical areas. In this context, some authors (Zsoldos et al., 2016) suggest that automatic processing of emotion may be preserved during aging, whereas deliberate processing (as PoFA performance in our study) is impaired.

### Differences in Emotion Recognition: The Influence of Gender

Many studies underline the advantage of women in decoding emotions (Thompson and Voyer, 2014; Sullivan et al., 2017; Olderbak et al., 2018). Females show an advantage over males, even from infancy. However, the studies do not always confirm that gender differences are maintained across the age span (Olderbak et al., 2018); for instance, a meta-analysis (Thompson and Voyer, 2014) showed that the largest sex differences were for teenagers and young adults, while other studies underlined that females performed better than males in recognizing emotions in middle and older age (Demenescu et al., 2014).

In our older adults, sample females seem to show significantly higher scores in anger recognition on PoFA compared to males. On total M-PoFA, females also seem to show more ability than males to recognize paired emotions. Therefore, females showed a pattern of emotion recognition that might suggest a tendency toward more “susceptibility” to anger and better emotions discrimination than males when faced with identical paired emotions in M-PoFA.

Some authors observed that identification of anger, sadness, and fear is easier when subjects explore the top half of the face, whereas disgust and happiness are better recognized when the bottom half of the face is explored. Identification of surprise is related both to the top and bottom half of faces (Calder et al., 2000; Sullivan et al., 2017; Birmingham et al., 2018). In our sample females seem to explore the top half of the face earlier than males and identify anger better than males. Therefore, we observed that, in exploring face to decode emotions, males fixated on the forehead later than females.

### Differences in Emotion Recognition: The Influence of Aging and Gender

Considering the effect of gender on emotion recognition separately within the YAG and the OAG, we observed that:

(a) In the YAG, there were no differences between males and females in emotion recognition, nor in exploration strategies or cognitive functions. If confirmed by a larger sample, this result might be in contrast with studies finding that young women’s emotion recognition exceeds that of young men (Sullivan et al., 2017) and with the meta-analysis of Thompson and Voyer (2014) showing that the largest sex differences were for teenagers and young adults. It might also be in contrast with previous Eye-tracking studies finding that young females spend more time looking at eyes compared with mouth relative to young males (Hall et al., 2010). On the other hand, our data might be in accordance with Sullivan et al. (2017) who, in considering only eyes and mouth areas, observed that young men and young women were similar in exploration strategies since they spent the same time looking at the eyes area.
(b) In the older adults, males seem to show significant lower scores compared to females in anger recognition, in M-PoFA total score for identical emotion pair and in total M-PoFA total correct scores. Therefore, older adult males showed a tendency toward a worse anger recognition. It is broadly known in literature that females are more accurate in labeling negative emotions and negative facial affects (e.g., anger, disgust) compared to males (Montagne et al., 2005; Scholten et al., 2005). Furthermore, our results are similar to many studies observing that older men have worse emotion recognition compared to older women (Ruffman et al., 2010; Campbell et al., 2014; Demenescu et al., 2014). Better anger recognition in females are reported in many studies that demonstrated the link between attention and emotion (Wrase et al., 2003; Hofer et al., 2006; Li et al., 2008; Gupta, 2012). Emotional and attentional processing involves activation of similar brain regions such as fronto-parietal regions, anterior cingulate cortex, inferior frontal gyrus, and anterior insula; brain areas that are also recruited in the processing of emotional information. In this context, the amygdala responds differentially to faces with emotional content, only when sufficient attentional resources were available to process those faces (Pessoa et al., 2002).

Considering exploration strategies, old males and old females differed for many variables in exploration strategies, and in particular the latter seem to look more at the forehead than the first. In contrast to the results from Sullivan et al. (2017), in our sample old males do not seem to look more at the mouth than old females, but seem to spend more time in fixation of right eye and left eye, while older adult females explore the frontal area more.

Including as covariates the eye-gaze fixations characteristics in which old males and old females differed, the difference in anger recognition between these two groups seems to disappear whereas the difference in recognition of identical pairs of emotions (M-PoFA) was maintained.

In other terms, between old males and old females, differences in anger recognition seem to be related to differences in exploration strategies. In addition to this, our study observed that differences in discrimination of same and different pairs of emotions seem to be unrelated to differences in exploration strategies. This result could allow us to hypothesize that older adult males may present a specific impairment of the individual emotion recognition pattern.

Nevertheless, our study presents some limitations. The small sample sizes did not allow a robust comparison between males and females. The high intensity of emotions represented in the PoFA and M-PoFA tasks may induce a ceiling effect in emotion recognition and may be unsuitable for sensitive and specific detection of differences in emotion recognition for healthy people. Perhaps an examination of the role of age and gender on facial emotion recognition in relation to neuropsychological functions and face exploration strategies would be advisable using dynamic stimuli and/or stimuli that depicted emotions at different intensities. Many studies have reported that more ecologically valid stimuli (i.e., dynamic) can improve emotion perception in younger adults (Ambadar et al., 2005), while older and-middle aged adults benefited from dynamic stimuli, but only when the emotional displays were subtle (Grainger et al., 2015). Thus, static photographs of intense expressions as in the PoFA test seem not to offer an appropriate instrument to detect older adult abilities in interpreting real life situations. Furthermore, correlation analyses between emotion recognition and quality of life need to be corrected for multiple testing in order to obtain more robust results. Further investigations should also include neuroimaging data that allow clarification for specific involvement of brain areas and a measure of mood that can offer a better explanation for the data of perceived quality of life.

## Conclusion

Our data confirms previous observations underlining that older adults are poorer in emotion recognition compared to younger adults, but there are no specific differences in anger, happiness, and disgust recognition. Moreover, in our study older subjects seem to show a diminished ability to recognize the similarity or the difference between two specific emotional patterns, regardless of their meaning.

As regards subjective quality of life, a better perception of some dimensions corresponds to worse recognition of emotions with negative valence. Furthermore, in the whole sample and particularly in older adults, satisfaction of life is also positively related to ability in recognizing the emotion pattern useful in discriminating different pairs of emotions, regardless of the meaning. Also in this task, we can underline the importance of psychological aspects such as well-being and motivation.

When controlled analyses for cognitive function and exploration strategies measures were conducted, in older people only the impairment of fear recognition is unrelated to cognitive functions and to exploration strategies. Finally, considering the differences between male and female, we preliminarily observed (in the general sample) that females seem to recognize anger and “discriminate” emotions better than males. After the separation of the sample into young males and young females, old males and old females, the latter showed a pattern of anger recognition that might be lower with respect to old females. Moreover, old males and old females differed in exploration strategies that appeared to determine the difference in anger recognition but not the difference in discrimination of same and different pairs of emotions.

## Data Availability Statement

The datasets generated for this study are available on request to the corresponding author.

## Ethics Statement

The studies involving human participants were reviewed and approved by Local Ethics Committee USL Tuscany South-Est, Grosseto, Italy. The patients/participants provided their written informed consent to participate in this study.

## Author Contributions

IR, MM, and NM designed the experiment. NM and MM collected the data. LA analyzed the data. LA, NM, and MM wrote the manuscript. All authors provided critical feedback and gave final approval of the version to be published.

## Conflict of Interest

The authors declare that the research was conducted in the absence of any commercial or financial relationships that could be construed as a potential conflict of interest.
