# Supplementary material for: Age and Gender Differences in Emotion Recognition
Source: Front Psychol. 2019 Oct 23;10:2371. doi: 10.3389/fpsyg.2019.02371 (PMC6819430; doi:10.3389/fpsyg.2019.02371)
Supplement: Supplementary file 1 [file Table_1.docx]

TABLE S1: Comparison between Younger and Older Adult Groups on neuropsychological variables and on eye movement variables.

|  | **Younger Adults Group** | **Older Adults Group** | **ANOVA** | | |
| --- | --- | --- | --- | --- | --- |
|  | **Mean (SE)** | **Mean (SE)** | **F** | **p value** | |
| **Cognitive functioning** | | | | |  |
| **Sustained attention** |  |  |  |  | |
| SART mean RT | 268.2 (20.6) | 406.9 (19.2) | 24.1 | 0.000* | |
| SART Omissions | 21.2 (10) | 54.3 (9.3) | 5.7 | 0.019* | |
| SART Commissions | 6 (1.2) | 10.8 (1.1) | 7.9 | 0.007* | |
| **Attentional shifting** |  |  |  |  | |
| TMT B - A | 33.7 (10) | 80.4 (9.4) | 11.4 | 0.001* | |
| **Frontal functioning** |  |  |  |  | |
| FAB | 17.3 (0.23) | 16.7 (0.21) | 3.6 | 0.06 | |
| **Visuo-spatial Long-term Memory** |  |  |  |  | |
| Corsi Supra Span Learning | 24.5 (1.2) | 18.1 (1.2) | 13.01 | 0.001* | |
| **Eye movements recording** | | | | | |
| Not_on_AOITime_to_first_fixation | 3996.4 (293.8) | 3667.3 (274.9) | 0.6 | 0.41 | |
| AOI_frontalTime_to_first_fixation | 293.8 (145.8) | 1945.4 (136.4) | 2.9 | 0.09 | |
| AOI_boccaTime_to_first_fixation | 1602.9 (138.9) | 1669.1 (129.9) | 3.1 | 0.08 | |
| AOI_distractorTime_to_first_fixation | 145.8 (200.4) | 3129.7 (187.9) | 1.9 | 0.16 | |
| AOI_globalTime_to_first_fixation | 1330.8 (165.9) | 2156.1 (155.2) | 0.03 | 0.85 | |
| AOI_osTime_to_first_fixation | 138.9 (102.49 | 1666.2 (95.8) | 3.5 | 0.06 | |
| AOI_odTime_to_first_fixation | 3515.3 (155.1) | 2061 (145.1) | 2.1 | 0.15 | |
| Not-on-AOI-Fixation-Length | 948.9 (69.7) | 625.3 (65.2) | 11.3 | 0.001* | |
| AOI_frontalFixation_Length | 1970.2 (108.4) | 1883.1 (101.4) | 0.3 | 0.56 | |
| AOI_boccaFixation_Length | 4383.1 (224.6) | 3990.8 (210.1) | 1.6 | 0.2 | |
| AOI_distractorFixation_Length | 1885.6 (159.6) | 2188.1 (149.3) | 1.9 | 0.1 | |
| AOI_globalFixation_Length | 4124.6 (266.8) | 3841.4 (249.6) | 0.6 | 0.44 | |
| AOI_osFixation_Length | 2886.6 (147.8) | 2606.7 (138.2) | 1.9 | 0.17 | |
| AOI_odFixation_Length | 1689.8 (120.7) | 1646.5 (112.9) | 0.06 | 0.79 | |
| Not-on-AOI-First-fixation-duration | 184.7 (8.4) | 161.7 (7.8) | 3.9 | 0.05* | |
| AOI_frontalFirst_fixation_duration | 321.3 (15.2) | 331.9 (14.2) | 0.2 | 0.61 | |
| AOI_boccaFirst_fixation_duration | 294,3 (11.8) | 291.4 (11.1) | 0.3 | 0.85 | |
| AOI_distractorFirst_fixation_duration | 300.1 (10.6) | 279.7 (9.9) | 1.96 | 0.16 | |
| AOI_globalFirst_fixation_duration | 299 (10.1) | 280.5 (9.5) | 1.73 | 0.19 | |
| AOI_osFirst_fixation_duration | 328.7 (15.9) | 327.6 (14.8) | 0.003 | 0.96 | |
| AOI_odFirst_fixation_duration | 300.6 (11.8) | 305.8 (11) | 0.1 | 0.74 | |
| Not_on_AOIFixation_Count | 3386.3 (257.1) | 2781.8 (240.5) | 2.94 | 0.09 | |
| AOI_frontalFixation_Count | 5284.6 (307.6) | 5314.9 (287.8) | 0.005 | 0.94 | |
| AOI_boccaFixation_Count | 11698.1 (606.1) | 11400.9 (567) | 0.1 | 0.72 | |
| AOI-distractor-Fixation-Count | 5244.1 (411) | 6417.57 (384.5) | 4.24 | 0.04* | |
| AOI_globalFixation_Count | 11051.3 (695.6) | 11215.5 (650.7) | 0.03 | 0.86 | |
| AOI_osFixation_Count | 7635.6 (387) | 7419.5 (362) | 0.16 | 0.68 | |
| AOI_odFixation_Count | 4751.3 (303.3) | 4662.1 (283.7) | 0.04 | 0.83 | |
| Not_on_AOIObservation_Length | 0.0 (0) | 0.0 (0) | 1.5 | 0.21 | |
| AOI-frontal-Observation-Length | 2465.6 (139.5) | 2843.3 (130.5) | 3.9 | 0.05* | |
| AOI_boccaObservation_Length | 5444.2 (142.9) | 5689.6 (133.6) | 1.5 | 0.21 | |
| AOI-distractor-Observation-Length | 2570.9 (229) | 3356.3 (214.2) | 6.14 | 0.016* | |
| AOI_globalObservation_Length | 5256.6 (233.9) | 5758.3 (218.8) | 2.4 | 0.12 | |
| AOI_osObservation_Length | 3620.5 (136.3) | 3746.9 (127.5) | 0.4 | 0.5 | |
| AOI_odObservation_Length | 2111.6 (113.4) | 2340.5 (106.1) | 2.1 | 0.14 | |
| Not_on_AOIObservation_Count | 0.0 (0) | 0.0 (0) |  |  | |
| AOI_frontalObservation_Count | 3792.1 (220.4) | 3354.03 (206.1) | 2.1 | 0.15 | |
| AOI_boccaObservation_Count | 2593.4 (116.7) | 2462.9 (109.2) | 0.66 | 0.41 | |
| AOI_distractorObservation_Count | 1822.7 (97.7) | 2016.06 (91.4) | 2.08 | 0.15 | |
| AOI_globalObservation_Count | 2001.8 (103) | 2066.6 (96.4) | 0.21 | 0.64 | |
| AOI_osObservation_Count | 3484.3 (173.7) | 3192.8 (162.4) | 1.5 | 0.22 | |
| AOI_odObservation_Count | 3736.3 (246.4) | 3296.4 (230.5) | 1.69 | 0.19 | |
| Not_on_AOIFixations_Before | 25790.8 (1812.2) | 21151.03 (1695.1) | 3.49 | 0.06 | |
| AOI_frontalFixations_Before | 8561.4 (669.5) | 10245.8 (626.3) | 3.37 | 0.07 | |
| AOI_boccaFixations_Before | 13810.3 (800.08) | 13149.9 (748.4) | 0.36 | 0.54 | |
| AOI_distractorFixations_Before | 25316.2 (1501.3) | 23382.1 (1404.4) | 0.88 | 0.35 | |
| AOI_globalFixations_Before | 22391.6 (1179.5) | 19773.7 (1104.3) | 2.62 | 0.11 | |
| AOI_osFixations_Before | 8649.3 (510.6) | 9672.8 (477.6) | 2.14 | 0.14 | |
| AOI_odFixations_Before | 9199.5 (828.6) | 10429.3 (775.08) | 1.17 | 0.28 | |
| Not_on_AOIParticipant_perc | 3996.4 (145.8) | 3667.3 (274.9) | 0.66 | 0.41 | |
| AOI_frontalParticipant_perc | 1602.8 (138.9) | 1945.4 (136.4) | 2.94 | 0.09 | |
| AOI_boccaParticipant_perc | 1330,8 (293.8) | 1669.1 (129.9) | 3.16 | 0.08 | |
| AOI_distractorParticipant_perc | 3515.3 (200.4) | 3129.7 (187.4) | 1.97 | 0.16 | |
| AOI_globalParticipant_perc | 2113.4 (165.9) | 2156.1 (155.2) | 0.03 | 0.85 | |
| AOI_osParticipant_perc | 1400.8 (102.4) | 1666.2 (95.8) | 3.57 | 0.06 | |
| AOI_odParticipant_perc | 1751.5 (155.1) | 2061.03 (145.1) | 2.12 | 0.15 | |
